# Heat Wave Beliefs and Behaviors in Southern Spain

**DOI:** 10.3390/ijerph22081196

**Published:** 2025-07-31

**Authors:** Aaron Metzger, Yuval Baharav, Peter Mitchell, Lilly Nichols, Breahnna Saunders, Alexis Arlak, Megan Finke, Megan Gottemoeller, Kurt Shickman, Kathy Baughman McLeod, Gregory A. Wellenius

**Affiliations:** 1 Marketing for Change, Alexandria, VA 22314, USA; 2 Adrienne Arsht-Rockefeller Foundation Resilience Center, Washington, DC 20005, USA; 3 Center for Climate and Health, Boston University School of Public Health, Boston, MA 02118, USA; 4 Climate Resilience for All, Washington, DC 20036, USA

**Keywords:** extreme heat, heat wave, heat safety, health behaviors, behavior change

## Abstract

Extreme heat is a pressing public health threat. This study assesses and describes the interrelationships between beliefs about heat waves, individuals’ precautionary behaviors during heat waves, and demographic factors. In May 2022, we surveyed 1051 residents (aged 25–90 years) in Southern Spain, a region that experiences frequent heat waves. We found that many participants engaged in heat wave avoidance (80.5%, e.g., spending more time indoors), impact reduction (63.7%, e.g., drinking more water), or prosocial behavior (31.6%, e.g., helping others). However, one in four (25.9%) respondents also indicated that they personally do not need to worry about heat waves. Heat wave beliefs and behaviors were modestly correlated with demographic characteristics. Individuals who view themselves as less vulnerable to heat-related health risks (“impervious” beliefs) were less likely to report altering their behavior during heat waves. Public health efforts aiming to change behavior during heat waves may anticipate “impervious” beliefs and demographic differences in risk perception and heat-related behaviors.

## 1. Introduction

Extreme heat events (EHEs, i.e., “heat waves”) pose a major and escalating threat to public health [1]. Heat waves are typically the deadliest meteorological hazard globally [2,3,4], accounting for an estimated 500,000 annual excess deaths worldwide [5]. In the summer of 2022, over 60,000 heat-related deaths occurred in Europe, with Spain experiencing one of the highest rates of heat-related mortality [6]. Extreme heat puts stress on multiple physiological systems with potentially deadly consequences including heart attacks, worsening symptoms among those with asthma and chronic obstructive pulmonary disease (COPD), kidney damage, and death due to heat stroke [7]. Extreme heat also contributes to significant non-fatal health effects including increasing the rate of emergency department (ED) visits, worsening mental health outcomes, and increasing the risk of pregnancy-related complications [8,9,10]. Heat is more dangerous to those with certain health conditions, those in certain age groups (e.g., children or the elderly), and those at particular risk of high exposure (e.g., outdoor workers). Additionally, individuals living in communities of lower socioeconomic means often experience greater exposure to extreme heat and more negative health outcomes from EHEs [11].

Several studies have identified heat wave safety behaviors and measured their prevalence in countries affected by extreme heat. For example, Sheridan (2007) measured seven “responses to heat waves” among individuals living in North American cities, including behaviors such as whether individuals drank more water than normal, sought out a cooler location, and behaved any differently in conditions of heat [12]. Lefevre et al. (2015) and Erens et al. (2021) assessed engagement in similar sets of heat wave safety behaviors in England, including spending more time indoors, closing curtains, drinking more cool fluids, limiting physical activity, and avoiding alcohol [13,14]. Ban et al. (2019) surveyed participants in Jinan, China, and documented positive associations between respondents’ reported concerns (about either weather or health risks) and their reported adaptive behaviors [15]. van Loenhout et al. (2021) surveyed participants in Tunisia, Georgia, and Israel with an open-ended question: “Are you familiar with some measures you can take to protect yourself from a heatwave?” [16]. Participant responses included behaviors such as increased fluid consumption, avoiding physical activity, adjusting diet, adjusting clothing, and staying inside. While there is considerable overlap in the heat wave safety behaviors measured across these studies, previous research has analyzed each heat wave safety behavior separately, rather than attempting to group behaviors based on the intended goal of the behavior in the face of heat waves (e.g., avoiding heat exposure vs. reducing health risk). In addition, little research has systematically measured engagement in prosocial heat wave safety behaviors aimed at helping others avoid the dangers of extreme heat.

In addition to engagement in safety behaviors, it is also important to consider an individual’s perceptions of and attitudes toward heat waves, since underestimation of the health dangers posed by extreme heat could reduce an individual’s engagement in heat wave protective behaviors [17,18,19]. Regarding heat wave perceptions and attitudes, some previous research has focused on general participant perceptions of whether heat waves posed a health risk to themselves, their family, and their community [20]. Other studies have measured whether individuals know what negative health symptoms may accompany heat waves and whether they know what groups of people may be most vulnerable to heat waves [16]. Researchers have also investigated whether participants believed heat wave safety behaviors would be effective at reducing negative health risks [13,14]. Less research has explored specific perceptions of heat waves and attitudes toward protective strategies, such as perceptions that heat waves are more dangerous than other natural disasters or that spending time outside during a heat wave increases the risks of heat stroke. Similarly, few studies have examined self-efficacy beliefs concerning heat wave risks, such as whether individuals believe they have the skills necessary to stay safe during heat waves.

Thus, the current evidence suggests the presence of a gap between the documented health risks related to extreme heat and individuals’ perception of those risks and behaviors during extreme heat events. Moreover, there is limited evidence regarding the connection between people’s perceptions of risk or demographic characteristics and their proclivity to change their behavior during extreme heat events. Finally, there is little information on how increasing extreme heat, demographics, and individual risk perception of heat impacts receptiveness to emerging government interventions aimed at reducing population risk.

To address these evidence gaps, we conducted a survey examining the prevalence of heat wave safety behaviors and heat wave beliefs in several municipalities in Southern Spain. We sought to (1) identify behaviors and beliefs related to extreme heat events in a region that experiences frequent heat waves and encompasses Seville, the hottest city in Europe, and (2) provide policy makers, health services, weather services, extreme heat early warning systems, and public health efforts with insights into the prevalence of different beliefs and behaviors, along with the characteristics of individuals who are likely (and unlikely) to take heat wave safety precautions.

## 2. Materials and Methods

### 2.1. Study Design and Procedures

**Figure 1:**
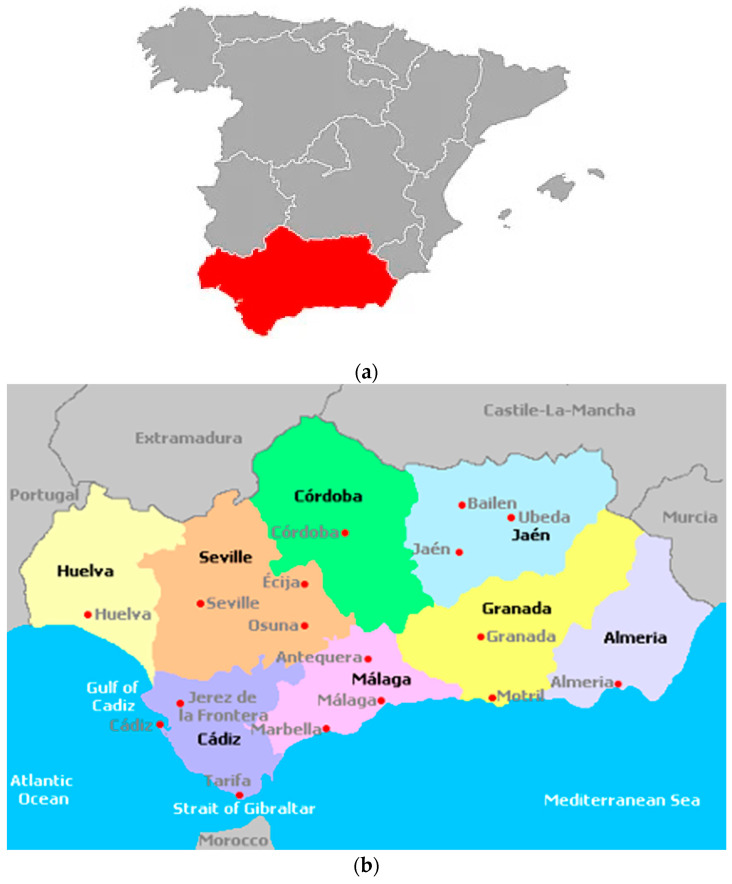
Maps of the regions of Spain included in the study:(**a**) the autonomous region of Andalusia in Southern Spain and (**b**) the municipalities of Cadiz, Cordoba, Huelva, Malaga, and Seville.

Between 4 and 18 May 2022 we distributed an online survey to a sample of adults aged 25 years to 90 years old who lived in the municipalities of Cádiz, Córdoba, Huelva, Málaga, and Seville within the autonomous region of Andalusia in Southern Spain (Figure 1). Individuals were sampled and recruited from an online panel that included respondents across urban and rural areas in the region. The survey was part of a larger research effort assessing beliefs and behavior regarding extreme heat events during the months leading up to heat wave season. The design of the survey and the methods for sampling respondents were similar to those in our previously published study conducted in the same geographic area [21].

### 2.2. Measures

#### 2.2.1. Heat Wave Beliefs

Participants used a 5-point Likert scale (strongly disagree, somewhat disagree, neither agree or disagree, somewhat agree, strongly agree) to indicate their level of agreement with seven specific beliefs about heat waves. Three items assessed how familiar participants were with potential health dangers of EHEs, three items measured self-efficacy beliefs about engaging in EHE-safe behaviors, and one item assessed support for government action to protect citizens from heat waves (See the Supplementary Material Survey S1 for a complete view of the survey). 

#### 2.2.2. Heat Wave Safety Behaviors

Individuals can engage in a range of behaviors to decrease the health risks posed by heat and avoid discomfort [4]. Avoidance heat wave behaviors are those used to reduce an individual’s exposure to heat, such as by spending more time indoors or adjusting work or leisure activities. In contrast, reduction heat wave behaviors are those used to lessen or mitigate the effects of extreme heat, such as by drinking more water or wearing different clothes. Finally, prosocial behaviors are those aimed at helping others reduce their exposure or risk, such as by warning others, discussing strategies for reducing health impacts, or directly helping others. Prosocial behaviors may contribute to increasing overall community safety and reducing health risks during EHEs [22]. Participants were asked, “Which, if any of the following, did you do during that heat wave?” and were presented with a checklist of risk avoidance (“Spent more time inside”, “Changed my leisure plants to avoid the high heat”, “Changed my work hours to avoid the high heat”, “Worked from home to avoid the high heat”, and “Found a place outside my home to stay cool”), risk reduction (“Drank more water than I usually do”, “Dressed differently to protect myself from the heat”, and “Changed what I eat”), and prosocial behaviors (“Warned others about the heat wave”, “Told others how to stay safe in the heat”, and “Helped someone else avoid the high heat”). Participants were instructed to check all that apply. Three response options (“Spent more time outdoors”, “More closely followed the forecast”, and “I did nothing differently than I usually do”) did not fit into the three behavior categories outlined above and were not included in the analysis of those behavior types.

#### 2.2.3. Air Conditioner (AC) Usage

Respondents were asked, “Which of the following means of avoiding heat do you have in your home?” Checklist response options included AC and ceiling fans, among other assets. Respondents indicating that they had an AC in their home were asked, “How often do you decide not to turn on your air conditioner on hot days because of the high price of electricity?”. The measure included four response options: 1. “I have completely stopped using my air conditioner due to high costs.”; 2. “I don’t use my air conditioner on most very hot days, even though I need it, due to the high price of electricity.”; 3. “I don’t use my air conditioner on some very hot days, even though I need it, due to the high price of electricity.”; and 4. “I use my air conditioner on very hot days even though it can be very expensive.”

#### 2.2.4. Participant Demographics

Participants reported age, gender, income, health status, city of residence, work conditions, and cooling access. Monthly income was reported on a 12-point scale with categories ranging from less than 570 euros to greater than 6000 euros per month. We assessed health status via a single question: “In the last twelve months, would you say that your state of health has been very good, good, fair, bad, very bad?” We used these demographic risk factors to identify how heat beliefs impact heat health behaviors specifically in populations who are more vulnerable to the effects of heat exposure including older individuals, those with chronic health conditions, those exposed to high heat at their place of work, and individuals with low socioeconomic status. We compared the sex and age distribution of respondents to the sex and age distribution of the population of Andalusia [23].

#### 2.2.5. Missing Data

The vast majority (97.5%) of respondents provided complete data on all variables. Missing data were singly imputed using mean imputation [24,25].

### 2.3. Analysis Strategy

We grouped participants into three age groups (25–44, 45–59, ≥60), three income groups (<1300 euros/month, 1300–2199 euros/month, ≥2200 euros/month), and two health status groups (good/very good health vs. bad/very bad/fair). We first examined descriptive characteristics (mean, SD, range) for the heat wave belief items and Pearson correlation coefficients between heat wave beliefs and demographic characteristics. Next, we calculated frequencies for the heat wave safety behaviors for behavior categories (risk avoidance, risk reduction, prosocial) as well as individual items.

Finally, we used linear regression models to quantify associations between demographic risk factors (gender, age, income, and health status), heat wave beliefs, and heat wave safety behaviors. The dependent variable in each model was the total number of reported avoidance, reduction, or prosocial behaviors.

We explored bivariable (i.e., unadjusted) associations between responses to each of the seven belief questions and the number of heat wave safety behaviors participants reported. Based on initial results, we fit additional regression models for each behavior category, including the following beliefs as predictors: “Spending more time outdoors puts me at greater risk of heat stroke.”; “Healthy people do not need to change their daily routine.”; and “I am a person who does not need to worry about heat waves.” These models were adjusted for age, sex, income, and health status. Analyses were conducted using SPSS v. 29 and a 2-sided *p*-value < 0.05 was considered statistically significant.

## 3. Results

Individuals were sampled and recruited from an online panel. A total of 1650 individuals were invited to participate and 1051 participants completed the online survey (63.7% response rate). Respondents ranged in age from 25 to 90 years old, with approximately half identifying as female (52.5%). Compared to the population of Andalusia, adults aged 30–59 years were somewhat overrepresented in the survey while adults 60 and older were underrepresented relative to the population of the region (Table 1). Most respondents reported a monthly income of 800–1550 (38.3%) or 1550–2700 (33.3%) euros and a majority of respondents (72.7%) reported that their health status was “good” or “very good”. The sample’s reported income was on average slightly lower than the average monthly income of Andalusia [26]. The distribution of self-reported health status was similar to that reported in previous studies of Spanish adults [21,27].

### 3.1. Heat Wave Beliefs

Over 80% of respondents agreed that spending time outside during heat waves increases their risk of heat stroke and that some heat waves are more dangerous than others (Table 2). However, 20% of respondents agreed (completely agree or agree) that healthy people do not need to change their daily routines during a heat wave, 66% agreed that heat waves pose a risk but are “manageable where I live”, and nearly 90% of participants agreed that they knew how to stay safe during heat waves. More than 25% of respondents reported that they, themselves, are not the type of person who needs to worry about heat waves.

**Figure 2:**
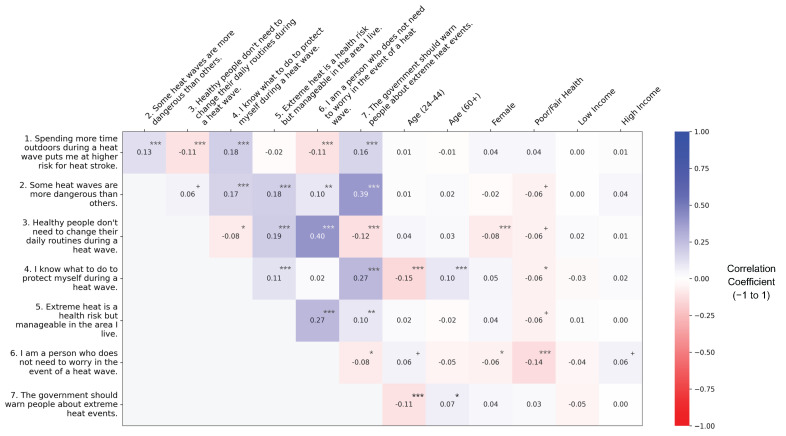
Point-biserial correlations between heat wave safety perceptions and attitudes and demographic risk factors. Southern Spain, 2022. + = *p* < 0.1, * *p* < 0.05, ** *p* < 0.01, *** *p* < 0.001.

Heat wave beliefs were only moderately correlated with each other and weakly correlated with demographic factors (Figure 2). Those that agreed that they personally do not need to worry during a heat event tended to also agree that healthy people do not need to change their routines during a heat wave (Pearson r = 0.40, *p* < 0.001) and that heat-related health risks are manageable where they live (r = 0.27, *p* < 0.001). Those that agreed that the government should warn people about extreme heat events tended to also agree that some heat waves are more dangerous than others (r = 0.39, *p* < 0.001) and that they know how to protect themselves during a heat event (r = 0.27, *p* < 0.001).

Compared to middle-aged adults (45–59), younger respondents (25–44) (r = −0.15, *p* < 0.001) were less likely and older respondents (60+) (r = 0.10, *p* < 0.001) more likely to agree that they knew what to do to stay safe during a heat wave. Younger respondents were also less likely than middle-aged adults to agree that the government should warn people about heat waves (r = −0.11, *p* < 0.001). Those who self-reported poorer health were less likely to agree than those who reported better health that they did not need to worry about heat waves (r = −0.14, *p* < 0.001). Income was not associated with heat wave beliefs.

### 3.2. Heat Wave Safety Behaviors

**Figure 3:**
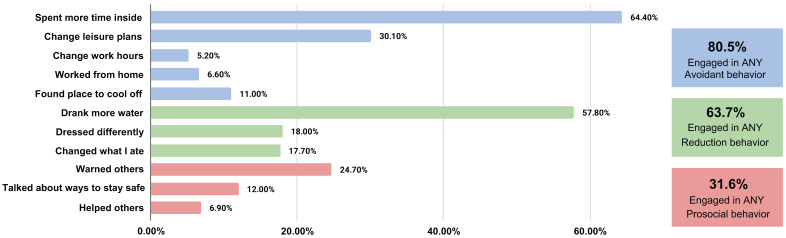
Percentage of reported engagement in heat wave safety behaviors. Southern Spain, 2022.

**Figure 4:**
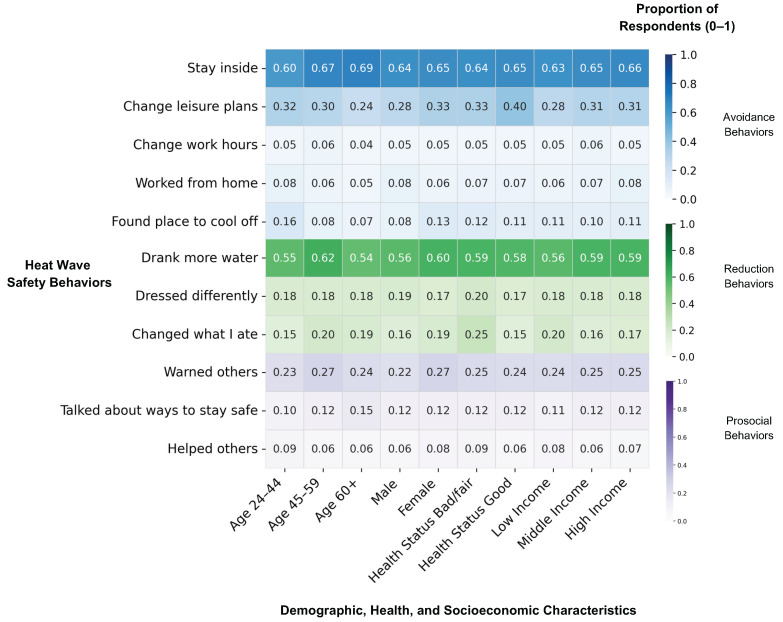
Heat map depicting the proportion of respondents reporting different heat wave safety behaviors by various demographic, health, and socioeconomic characteristics. Southern Spain, 2022. Darker color represents a higher proportion of respondents from a given category. The different types of heat wave safety behaviors (avoidance, reduction, prosocial) are represented by different colors as shown on the right axis.

The majority of respondents engaged in one or more heat wave avoidance (80.5%) or risk reduction behaviors (63.7%) (Figure 3). Only two behaviors were engaged in by over 50% of the sample: spent more time indoors (64.4%) and drank more water (57.8%). Almost a third (30.1%) of respondents adjusted their leisure activities, but only a small fraction adjusted their work hours (5.2%) or looked for a place outside of their home to stay cool (11.0%). Almost a third (31.6%) of participants engaged in prosocial behaviors, primarily warning others about heat (24.7%). The proportion of participants reporting specific behaviors was similar across subgroups of age, sex, health, and income (Figure 4).

We used hierarchical linear regression models to identify predictors of heat wave safety behaviors, mutually adjusting for gender, age, income, and health status. Results from these models including standardized regression coefficients and *p* values can be found in Table 3. Younger adults were less likely than middle-aged adults to engage in risk reduction behaviors in models adjusted for gender, income, and health status. On the other hand, participants reporting being in fair or poor health were more likely to engage in risk reduction behaviors. Age, sex, income, and health status were not predictive of other safety behaviors.

Individuals that agreed with the statement “I am a person who does not need to worry in case of a heat wave” were less likely to participate in heat avoidance, risk reduction, and prosocial behaviors in models adjusted for age, sex, income, and health status. Similarly, individuals that agreed with the statement, “Healthy people do not need to change their daily routines during a heat wave”, were less likely to participate in heat avoidance, risk reduction, and prosocial behaviors in adjusted models. Finally, participants who agreed that spending time outdoors during heat waves increased the risk of heat stroke were more likely to participate in heat avoidance behaviors. Overall, these associations were small to moderate in magnitude. For instance, the risk reduction behavior regression coefficient of b = −0.14 indicates that compared to a person who strongly agreed with the statement “Healthy people don’t need to change their daily routines during heat waves”, a person who strongly disagreed reported approximately 0.56 fewer heat wave reduction behaviors.

### 3.3. Air Conditioner Use

Most (70.6%) respondents indicated that they had some form of AC in their home. Among those who own an AC, 65.1% indicated that they use their AC on hot days regardless of expense, while the remaining 34.9% limited AC use to some degree including 5.5% who stopped using their AC completely due to cost. Other participants who reported reducing their AC due to expense either turned off their AC on some (14.5%) or most (14.9%) very hot days due to the high price of electricity.

A similar regression model to those described above was fit to predict AC usage on hot days among participants who reported owning an AC (n = 724). The dependent variable in this model was frequency of AC use on hot days (despite costs). Individuals were more likely to use their AC more frequently (despite costs) if they reported high monthly incomes (b = 0.13, SE = 0.06, *p* = 0.03) or had poorer health (b = 0.13, SE = 0.06, *p* = 0.02). Individuals were more likely to report reduced AC use on hot days due to concerns about the costs if they were younger (b = −0.12, SE = 0.05, *p* = 0.02) or reported lower incomes (b = −0.13, SE = 0.06, *p* = 0.03). AC use was not associated with heat wave perceptions or attitude variables.

## 4. Discussion

We conducted a survey in Southern Spain during the time frame immediately prior to the expected heat wave season to assess the prevalence of, and interrelationships between, beliefs about heat waves and engagement in heat avoidance, risk reduction, and prosocial heat wave safety behaviors. The majority of participants recognized that periods of extreme heat can harm people’s health, and large proportions of participants also expressed confidence that heat health risks were manageable, did not pose an important risk for healthy people, or did not apply to them as individuals. The large majority of participants agreed that governments should warn people about extreme heat events. Beliefs about heat waves varied weakly across demographic groups. A primary finding of the current study is that individuals with lower belief in heat wave risks, or who do not view heat waves as being dangerous to their own health, (i.e., “impervious” beliefs) are somewhat less likely to engage in heat safety behaviors.

### 4.1. Heat Wave Beliefs

These results are broadly consistent with previous research from the United States, United Kingdom, China, and elsewhere finding that heat waves are not generally recognized by the public as a serious threat to health, although with substantial variation across studies [12,13,15,16]. While most respondents in our study recognized that heat exposure during heat waves is associated with adverse health impacts, and that some heat waves are more dangerous than others, roughly a quarter of respondents believe that healthy people face little risk from heat waves and that they personally do not need to worry about heat waves. These beliefs may be most dangerous if held by vulnerable or heat-exposed populations, including pregnant individuals and caregivers for infants and young children [28], heat-exposed workers who face economic- and health-related losses [29], athletes [30], individuals taking certain prescription and over-the-counter medications that inhibit sweating or thermoregulation [28], older adults, the unhoused, and individuals with chronic illness [22,28,29,30,31]. Controlling for participant demographics, including income, health status, and age, individuals with “impervious” beliefs were less likely to report taking precautions during extreme heat in all three behavior categories: avoidance, protective, and prosocial. These findings suggest that increased education is still needed to further raise public awareness about the health risks of heat waves, with particular emphasis on helping individuals recognize that no one is immune or impervious to these health risks.

### 4.2. Heat Wave Safety Behaviors

Previous studies support the notion that during heat waves a large majority of individuals engage in at least some heat avoidance or risk reduction activities [12,13,14,16,17]. Similarly, in this survey we found that a large majority of participants reported engagement in one or more heat avoidance or risk reduction behaviors, with only modest variation across demographic subgroups. These findings suggest that at least some heat avoidance behaviors (primarily spending more time indoors and/or modifying leisure plans) and risk reduction behaviors (primarily drinking more water) are already commonly practiced across this population. We found modest evidence that younger adults engaged in fewer risk reduction behaviors than middle-aged adults, and that those in fair/poor health engaged in more reduction behaviors.

Heat alerts or similar messaging from governments has been associated, in some cases, with increased engagement in safety behaviors [14]. However, the effectiveness of heat alerts and other broad messaging in reducing health risks at a population scale remains uncertain. For example, a survey across four North American cities found that fewer than half of participants reported changing their behavior on heat wave days despite near universal awareness of the extreme heat event [12]. This finding is consistent with population-scale research in the US that fails to find an association between the issuing of heat alerts and reductions in mortality on hot days [32,33]. On the other hand, a previous study in this same region of Spain suggests that increased awareness (in that case, by assigning a name to a particularly severe heat wave and the resulting media coverage) found that those that could recall the named heat wave were more likely to engage in protective behaviors [21]. These findings highlight the lack of one-size-fits-all solutions to encouraging individuals to engage in more heat safety behaviors during periods of extreme heat.

Nearly a third of respondents reported engagement in prosocial behaviors, which is substantially lower compared to the proportion that reported engagement in avoidance and reduction behaviors. Importantly, individuals holding “impervious” beliefs engaged in fewer prosocial behaviors, suggesting these individuals may be having fewer conversations about heat wave risks or efforts to help others during extreme heat events. While avoidance and reduction behaviors are important for protecting individuals from the dangers of heat waves, engagement in prosocial activities may also serve a protective role at the community level. Previous research has examined community-level implications of prosocial behavior during emergency events, such as the COVID-19 pandemic [34], and finds that prosocial behaviors during emergencies can be protective for disadvantaged communities. Warning others, discussing ways to stay safe, and helping others before and during EHEs may raise community-level awareness, encourage more people to take precautions, and ultimately reduce negative health outcomes.

Individuals with lower incomes tended to report less access to AC and being less likely to run their AC unit due to prohibitive cost. This finding is consistent with previous research, which has consistently found that socioeconomic status is a persistent barrier to residential air conditioning [12,35].

### 4.3. Limitations

Findings from the current study should be interpreted in light of limitations. First, findings are limited to Southern Spain. The sample characteristics were generally representative of the municipalities in Southern Spain that were included in the study, but there were proportionally more middle-aged participants (aged 45–54) compared to older adults (60 and older). The survey was administered in the spring (May 2022) as an intentional effort to assess heat wave beliefs and behavior in the season immediately prior to the hotter summer months. However, participants reported on their behavior during a heat wave from the previous summer (summer 2021), so responses may have been confounded by recall biases. Future research should consider measuring the frequency of engagement in these behaviors rather than a yes/no checklist. The survey did not allow respondents to report on whether they lived in a rural, urban, or suburban environment. Both beliefs about heat waves and precautionary behavior taken by individuals during extreme heat events may differ for urban and rural locations, and future research should explore this possibility. Additionally, while we were able to capture heat health beliefs and behaviors in some vulnerable groups such as the elderly, low-income individuals, heat-exposed workers, and those with chronic health conditions, due to survey length restrictions we were unable to capture other vulnerability factors including housing quality and impacts on pregnant women and children. The survey did include a question that assessed whether respondents typically worked inside, outside, or both. Individuals who had flexibility in their work schedules (worked both inside and outside) were more likely to engage in all three heat wave safety behaviors, but inclusion of work environment did not impact any of the key findings or model parameters. Future research should continue to explore how different types of inside and outside work schedules affect heat wave beliefs and behavior. Finally, associations between participants’ EHE safety behaviors and beliefs were examined cross-sectionally, so future research should utilize longitudinal data to examine predictive associations over time.

## 5. Conclusions

This study has multiple implications for public health policy, particularly the continued importance of raising awareness about the dangers of heat and encouraging heat wave safety behaviors across demographics. The findings suggest that demographic characteristics may be associated with attitudes toward heat waves and engagement in different types of heat wave safety behaviors. These insights may be useful for informing more effective public messaging about heat. As “impervious” beliefs were associated with lower engagement in avoidance, reduction, and prosocial behaviors, individual-level heat risk assessments could consider screening for beliefs that “heat isn’t dangerous to me” or other “impervious” beliefs. Moreover, these findings suggest that in addition to avoidance and reduction behavior recommendations, public messaging could also encourage and prioritize prosocial behaviors, which had the lowest levels of engagement in this sample. Recommendations that include specific protective strategies for avoiding the heat (when/where possible), reducing extreme heat impacts, and identifying the signs and symptoms of heat stroke might be more readily embraced by individuals in occupations or conditions where heat exposure is unavoidable [22]. Additionally, those with poorer health believed that they were at higher risk from heat but were less likely to agree that they knew what to do to stay safe. Yet, those with poor health status were also more likely to engage in reduction behaviors. Thus, poor health status individuals may benefit from health care providers and public health campaigns placing additional emphasis on heat risk awareness and education. Low belief in heat wave risks and belief that heat waves are not dangerous for oneself—or “impervious” attitudes—were associated with lower engagement in all three heat wave safety behavior categories. “Impervious” attitudes may also reduce the efficacy of messaging designed to increase safe heat wave behaviors. People who believe that heat does not pose a threat may be less likely to heed warnings about the dangers of upcoming heat waves. Future research should assess whether messaging heat as a threat to everyone, rather than only vulnerable groups, decreases impervious beliefs and improves heat health behaviors in participants. Efforts to address the thermal safety and comfort of homes should address the economic barriers to both owning and running AC units.

## Figures and Tables

**Table 1:** Characteristics of study participants (n = 1051).

	% of Respondents	% of Population of Andalusia
Gender		
Female	52.5%	50.8%
Age		
20–29	9.0%	10.8%
30–39	23.0%	12.4%
40–49	30.6%	15.9%
50–59	24.0%	15.5%
60–69	11.0%	12.2%
70–79	2.0%	8.1%
80–89	0.2%	4.1%
90+	0.1%	0.9%
Monthly Income (Euros)		
<800	9.1%	N/A
800–1550	38.3%	N/A
1550–2700	33.3%	N/A
>2700	19.3%	N/A
AVERAGE monthly income		2426
Health Status		
Very Good	15.6%	N/A
Good	57.1%	N/A
Fair	23.7%	N/A
Poor/Very Poor	3.5%	N/A

Symbol: N/A = Data not available. Note: Andalusia data from the Instituto Nacional de Estadística, Madrid [23,26].

**Table 2:** Descriptive statistics for heat wave belief measures.

		Mean	SD	Range	Partially/Completely Agree
Perceptions about Heat Waves					
	Spending more time outdoors during a heat wave puts me at higher risk for heat stroke.	4.28	1.23	1–5	86.7%
	2. Some heat waves are more dangerous than others	4.19	1.00	1–5	81.1%
	3. Healthy people don’t need to change their daily routines during a heat wave.	2.28	1.23	1–5	21.3%
Self-efficacy beliefs about heat waves					
	I know what to do to protect myself during a heat wave.	4.33	0.74	1–5	89.8%
	2. Extreme heat is a health risk but manageable in the area I live.	3.62	1.20	1–5	63.8%
	3. I am a person who does not need to worry in the event of a heat wave.	2.56	1.22	1–5	25.9%
Government responsibility to protect citizens					
	The government should warn people about extreme heat events.	4.61	0.75	1–5	91.6%

**Table 3:** Regression models with each category of heath wave protective behaviors predicted by participant characteristics and attitudes toward heat waves.

	Heat Avoidance Behaviors		Risk Reduction Behaviors		Prosocial Behaviors	
Model STEP 1	β	*p*	β	*p*	β	*p*
Gender (Female) ^+^	0.05	0.12	0.03	0.30	0.05	0.11
Age: younger (25–44) ^++^	0.02	0.50	−0.07	0.047	−0.02	0.59
Age: older (60+) ^++^	−0.03	0.31	−0.04	0.19	−0.01	0.81
Monthly Income: low ^#^	−0.04	0.27	−0.01	0.74	−0.01	0.88
Monthly Income: high ^##^	0.03	0.40	0.02	0.42	0.01	0.70
Health Status: poor/fair	0.05	0.16	0.07	0.029	0.02	0.49
Model STEP 2						
^ Time outdoors increases risk	0.11	<0.001	0.01	0.93	0.01	0.98
^^ I don’t need to worry	−0.07	0.024	−0.07	0.044	−0.06	0.064
^^^ Healthy people don’t need to change	−0.09	0.008	−0.19	<0.001	−0.13	<0.001
R^2^	0.04		0.04		0.01	

Symbols: ^+^ Males are the comparison group. ^++^ Middle-aged adults (45–59) are the comparison group. ^#^ Income = Under 1300 euros per month; ^##^ Income = 2200 euros per month or more. The following symbols denote specific survey questions: ^ “Spending more time outdoors during a heat wave puts me at higher risk for heat stroke”, ^^ “I am a person who does not need to worry in case of a heat wave”, ^^^ “Healthy people do not need to change their daily routines during a heat wave”. β = standardized regression coefficient.

## Data Availability

Analytic data have been uploaded to the CAFE Collection of the Harvard Dataverse platform and can be found here: https://dataverse.harvard.edu/dataset.xhtml?persistentId=doi%3A10.7910%2FDVN%2FAKNUWF&version=DRAFT (accessed on 28 July 2025).

## Supplementary Materials

The following supporting information can be downloaded at https://www.mdpi.com/article/10.3390/ijerph22081196/s1; Survey S1: English Language Version Survey Instrument.

## Abbreviations

The following abbreviations are used in this manuscript:

EHEs	Extreme Heat Events
COPD	Chronic Obstructive Pulmonary Disease
ED	Emergency Department
AC	Air Conditioning
