# Supplementary material for: Heat Wave Beliefs and Behaviors in Southern Spain
Source: Int J Environ Res Public Health. 2025 Jul 31;22(8):1196. doi: 10.3390/ijerph22081196 (PMC12386305; doi:10.3390/ijerph22081196)
Supplement: Supplementary file 1 [file ijerph-22-01196-s001.zip › ijerph-3663399-supplementary.pdf]

SUPPLEMENTARY MATERIALS

for

Heat Wave Beliefs and Behaviors in Southern Spain

Aaron Metzger [1], Yuval Baharav [2], Peter Mitchell [1], Lilly Nichols [2,3], Breahnna Saunders [1], Alexis Arlak [3], Megan Finke [3], Megan Gottemoeller [1], Kurt Shickman [2], Kathy Baughman McLeod [2,4] and Gregory A. Wellenius [3,*]

[1] Marketing for Change, Alexandria, VA , 22314, USA; aaron.metzger@forchange.agency (A.M.); peter.mitchell@forchange.agency (P.M.); breahnna.saunders@forchange.agency (B.S.); megan.gottemoeller@m4c.co (M.G.)

[2] Adrienne Arsht-Rockefeller Foundation Resilience Center at the Atlantic Council, Washington, DC 20005, USA; baharavy@gmail.com (Y.B.); alilly@bu.edu (L.N.); kurtshickman@gmail.com (K.S.); kathy@climateresilience.org (K.B.M.)

[3] Center for Climate and Health, Boston University School of Public Health, Boston, MA 02118,, USA; aarlak@bu.edu (A.A.); mfinke17@bu.edu (M.F.)

[4] Climate Resilience for All, Washington, DC 20036, USA

* Correspondence: wellenius@bu.edu

Survey S1: *English Language Version Survey Instrument*

Screens

1. How old are you? [###]
    a. [Terminate if under 25]

2. Where do you live?
    a. Almería [Terminate]
    b. Cádiz
    c. Córdoba
    d. Granada [Terminate]
    e. Huelva
    f. Jaén [Terminate]
    g. Málaga
    h. Sevilla
    i. Somewhere else [Terminate]

3. Where did you live last summer?
    a. The same place as I do now
    b. Another place in Andalucia
    c. Some place outside Andalucia [Skip #4, ask #4-ALT instead]

Awareness (Part 1)

4. Thinking back to last summer, how many heat wave warnings occurred in your area? (If you're not sure, take your best guess) [pull-down, None to 15]
    a. #4 -ALT (for those who answered c to #3 only) - When did you move to Andalucia? [Month / Year]

5. When was the last time you recall hearing about a heat wave warning in your area? [pull-down]
    a. Before 2021
    b. May 2021
    c. June 2021
    d. July 2021
    e. August 2021
    f. September 2021
    g. Earlier this year
    h. I have never heard a heat wave warning [skip #6, and use ALT-#7]

6. Thinking back to that last heat wave, when did you first learn it was a heat wave?
    a. Two or more days before the heat wave began
    b. A day before the heat wave began
    c. The first day of the heat wave
    d. During the heat wave, but after the first day
    e. Only after the heat wave was over

7. Do you remember whether any of the heat waves last summer were named?

[if yes, then] What was the name of the heat wave you remember?

Behavior

8. Which, if any of the following, did you do during that heat wave? Check all that apply. [randomize order]

[ALT-#7: Which of the following did you do the last time you experienced a heat wave or extremely hot temperatures?"]

   a. Spent more time outdoors
   b. Spent more time indoors
   c. Changed my leisure plans to avoid the high heat
   d. Changed my work hours to avoid the high heat
   e. Worked from home to avoid the high heat
   f. Helped someone else avoid the high heat
   g. Drank more water than I usually do
   h. Warned others about the heat wave
   i. Told others how to stay safe in the high heat
   j. Found a place outside my home to stay cool
   k. More closely followed the forecast
   l. Dressed differently to protect myself from the heat
   m. Changed what I eat
   n. I did nothing differently than I usually do

9. Four typical glasses of water equal one liter. Roughly how many glasses of water did you drink on an average day during that last heat wave? [##]

10. How many glasses of water do you drink on an average day when there is no heat wave? [##]

11. During the worst heat wave last summer, how often were you able to change your plans to avoid the high heat?
    a. Never able to avoid the high heat
    b. Sometimes able to avoid the high heat
    c. Able to avoid the high heat about half the time
    d. Able to avoid the high heat most of the time
    e. Always able to avoid the high heat

Attitudes and Awareness (Part 2)

Please describe how much you agree or disagree with the following statements.

12. Healthy people do not need to alter their routine during a heat wave.
    a. Strongly agree
    b. Somewhat agree'
    c. Neither agree or disagree
    d. Somewhat disagree
    e. Strongly disagree

13. Heat waves kill more people in Spain than any other natural hazard, including floods, torrential rains and forest fires.
    a. Strongly agree
    b. Somewhat agree’
    c. Neither agree or disagree
    d. Somewhat disagree
    e. Strongly disagree

14. Spending time outdoors during a heat wave can put me at a significant risk of suffering a heat stroke.
    a. Strongly agree
    b. Somewhat agree’
    c. Neither agree or disagree
    d. Somewhat disagree
    e. Strongly disagree

15. I know what to do to stay safe during a heat wave.
    a. Strongly agree
    b. Somewhat agree’
    c. Neither agree or disagree
    d. Somewhat disagree
    e. Strongly disagree

16. I know how to help others during a heat wave.
    a. Strongly agree
    b. Somewhat agree’
    c. Neither agree or disagree
    d. Somewhat disagree
    e. Strongly disagree

17. Most people alter their routine to ensure their own safety during a heat wave.
    a. Strongly agree
    b. Somewhat agree’
    c. Neither agree or disagree
    d. Somewhat disagree
    e. Strongly disagree

18. I am not the kind of person who needs to worry about heat waves
    a. Strongly agree
    b. Somewhat agree’
    c. Neither agree or disagree
    d. Somewhat disagree
    e. Strongly disagree

19. Some heat waves are more threatening than others.
    a. Strongly agree
    b. Somewhat agree’
    c. Neither agree or disagree
    d. Somewhat disagree

e. Strongly disagree

20. The government should warn people about the most extreme heat waves.
    a. Strongly agree
    b. Somewhat agree’
    c. Neither agree or disagree
    d. Somewhat disagree
    e. Strongly disagree

21. Extreme heat is a dangerous but manageable health risk in my area.
    a. Strongly agree
    b. Somewhat agree’
    c. Neither agree or disagree
    d. Somewhat disagree
    e. Strongly disagree

Demographics

22. In the last twelve months, would you say that your state of health has been very good, good, fair, bad, very bad?
    a. Very good
    b. Good
    c. Fair
    d. Bad
    e. Very Bad[Text Wrapping Break]
23. Do you suffer or have you ever suffered from any of the following conditions? (Select all that apply).
    a. Heart conditions or coronary disease
    b. Respiratory problems (such as asthma and lung disease)
    c. Hypertension
    d. Diabetes
    e. Mental illness
    f. Drug or alcohol addiction
    g. None of these

24. Where do you work most of the time?
    a. Indoors, in an office
    b. Indoors, in my home
    c. Outdoors
    d. Both outdoors and indoors depending on the day
    e. Somewhere else. Please describe: [open]

25. Which of the following do you have in your home or apartment? (Select all that apply)
    a. Air conditioning
    b. Ceiling fans
    c. Window fans
    d. Other fans
    e. Shades or curtains to block the sun

f. Shaded film on the windows

Skip to 26, unless 24-a is chosen

26. How often do you decide not to operate your air conditioning on hot days due to the high cost of electricity?
    a. I have stopped using air conditioning completely due to the cost
    b. I don't use air conditioning on most hot days, even though I need it, because of the cost
    c. I don't use air conditioning on some hot days, even though I need it, because of the cost
    d. I use my air conditioning on hot days even though it can be expensive

27. Which of the following intervals best represents the net monthly income of your entire household, after deductions for taxes, Social Security, etc.?
    a. Less than 570 euros
    b. From 570 to less than 800 euros
    c. From 800 to less than 1050 euros
    d. From 1,050 to less than 1,300 euros
    e. From 1,300 to less than 1,550 euros
    f. From 1,550 to less than 1,800 euros
    g. From 1,800 to less than 2,200 euros
    h. From 2,200 to less than 2,700 euros
    i. From 2,700 to less than 3,600 euros
    j. From 3,600 to less than 4,500 euros
    k. From 4,500 to less than 6,000 euros
    l. From 6,000 euros onwards
